# The complete chloroplast genome of *Pterospermum kingtungense*, a Critically Endangered species

**DOI:** 10.1080/23802359.2018.1524271

**Published:** 2018-10-27

**Authors:** Zefu Wang, Xingxing Mao, Xiaoyue Yang, Lushui Zhang

**Affiliations:** a Ecological Security and Protection Key Laboratory of Sichuan Province, Mianyang Normal University, Mianyang, China;; b Key Laboratory of Bio-Resource and Eco-Environment of Ministry of Education, College of Life Sciences, Sichuan University, Chengdu, China

**Keywords:** Complete chloroplast genome, *Pterospermum kingtungense*, phylogenetic analysis

## Abstract

*Pterospermum kingtungense* (*Pterospermum*, Sterculiaceae) is a Critically Endangered species. In this study, we reported a complete chloroplast genome of *P. kingtungense* based on high-throughput sequencing data. The size of chloroplast genome was 162,929 bp, including a large single-copy region (LSC: 91,535 bp) and a small single-copy region (SSC: 20,464 bp), separated by a pair of inverted repeats (IRa and IRb: 25,465 bp). The genome encoded 126 genes in total, including 81 protein-coding genes, 8 ribosomal RNA genes, and 37 transfer RNA genes. The overall GC content of the *P. kingtungense* chloroplast genome is 36.39%. The phylogenetic tree showed *P. kingtungense* clustered together with the family Sterculiaceae.

*Pterospermum kingtungense* (*Pterospermum*, Sterculiaceae) is a Critically Endangered species. In the latest IUCN red list (http://www.iucnredlist.org/search), it is listed as Critically Endangered B1 + 2c, C2a. *Pterospermum kingtungense* could be used to make furniture, while root of which is also used as a traditional Chinese medicine. However, there is still not enough genetic information about such an endangered species. In this study, we reported the chloroplast genome of *P. kingtungense*. The annotated chloroplast genome has been submitted to GenBank under the accession number of MH606238.

The fresh leaves of a wild *P. kingtungense* plant were sampled from Dehong in Yunnan Province, China (97°32’E, 24°26’N). Voucher specimen of the species was stored in the Ecological Security and Protection Key Laboratory of Sichuan Province, Mianyang Normal University (Sichuan, China). We isolated its genomic DNA with the DNAsecure Plant Kit (TIANGEN). A paired-end library with the insert size of 350 bp was constructed and sequenced with the Hiseq4000 Platform (Illumina, USA). We obtained ∼10G high-quality paired-end reads for the subsequently analysis. Then, we mapped the raw reads to the reference, a complete chloroplast genome of *Theobroma cacao* (NC_014676), using the software Bwa (Li 2013) and Samtools (Li et al. 2009). We used the mapped reads to assemble the genome with the software NOVOPlasty v2.5.9 (Dierckxsens et al. 2017). After filling the gaps with GapCloser (Luo et al. 2012), a complete genome was then generated by the software Genious v 11.1.14 (Kearse et al. 2012). Finally, we obtained a chloroplast genome of *P. kingtungense* with a size of 162,929-bp. We annotated the genome with the software Plann (Huang and Cronk 2015) and Sequin (NCBI website).

The complete chloroplast genome of *P. kingtungense* has a typical quadripartite structure, comprising of two inverted repeats (IRA and IRB, 25,465 bp), separated by a large single-copy region (LSC, 81,935 bp) and a small single-copy region (SSC, 20,464 bp), respectively. GC content of the complete chloroplast genome, LSC, SSC, and IRs are 36.39%, 33.93%, 31.07%, and 42.95%, respectively. Genome annotation reveals a total of 126 genes, including 81 protein-coding genes (PCG), 37 transfer RNA (tRNA) genes, and 8 ribosomal RNA (rRNA) genes. Among these, 17 genes (6 PCGs, 7 tRNA genes, and 4 rRNA genes) are duplicated in the IR regions.

To investigate the phylogenetic position of *P. kingtungense*, we reconstructed a phylogenetic tree with the complete chloroplast genomes of 12 species including *P. kingtungense*. The sequences were aligned with the software MAFFT (Katoh and Standley 2013) and the phylogenetic tree was constructed using the software RaxML (Stamatakis 2014) with 100 bootstrap replicates. The phylogenetic tree showed all Malvales species clustered together with strongly support, and *P. Kingtungense* was closely related to the family Sterculiaceae (Figure 1).

**Figure 1.**
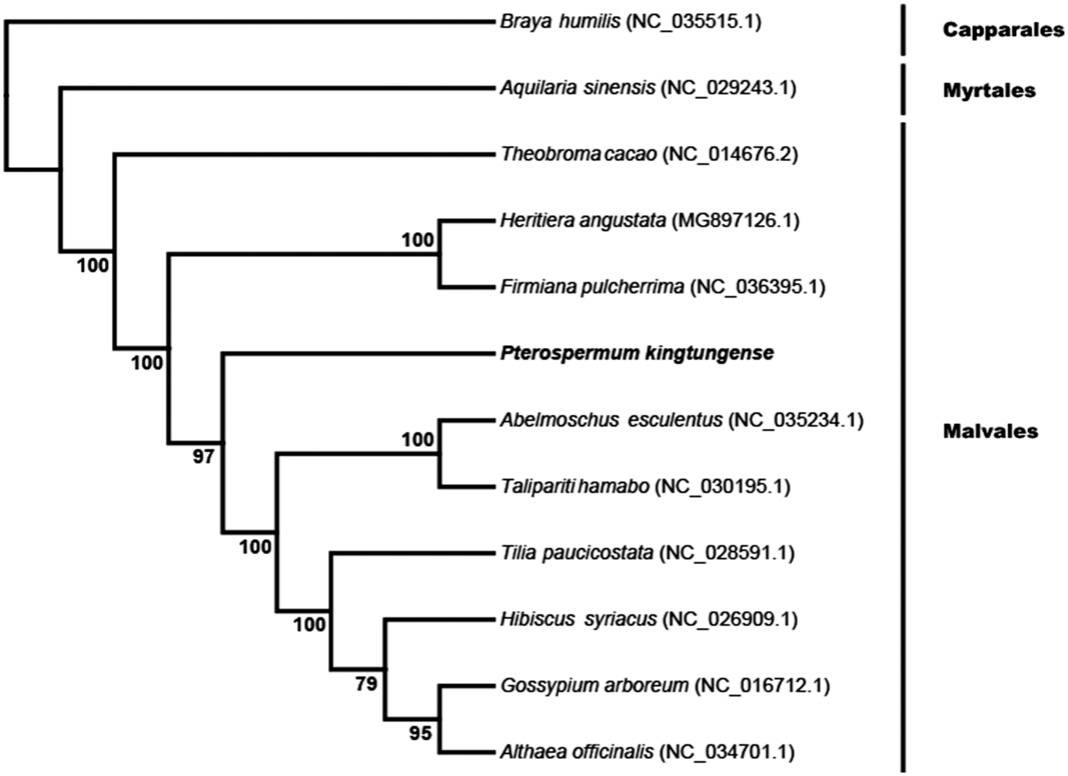
Phylogenetic tree based on the complete chloroplast genome sequences of Pterospermum kingtungense and other 11 species. Numbers in the nodes are the bootstrap values from 100 replicates.

Above all, we provide the valuable genomic information of *P. kingtungense*, and our findings could help us facilitate the identification, conservation, and utilization of this extremely endangered species.

## References

[CIT0001] Dierckxsens N, Mardulyn P, Smits G 2017 Novoplasty: de novo assembly of organelle genomes from whole genome data. Nucleic Acids Res. 45:e18. 10.1093/nar/gkw955 PMC5389512 28204566

[CIT0002] Huang DI, Cronk QCB 2015 Plann: a command-line application for annotating plastome sequences. Appl Plant Sci. 3:1500026. 10.3732/apps.1500026 PMC4542940 26312193

[CIT0003] Katoh K, Standley DM 2013 MAFFT multiple sequence alignment software version 7: improvements in performance and usability. Mol Biol Evol. 30:772–780. 23329690 10.1093/molbev/mst010 PMC3603318

[CIT0004] Kearse M, Moir R, Wilson A, Stones-Havas S, Cheung M, Sturrock S, Buxton S, Cooper A, Markowitz S, Duran C, et al. 2012 Geneious Basic: an integrated and extendable desktop software platform for the organization and analysis of sequence data. Bioinformatics. 28:1647–1649. 22543367 10.1093/bioinformatics/bts199 PMC3371832

[CIT0005] Li H 2013. Aligning sequence reads, clone sequences and assembly contigs with bwa-mem. arXiv:1303.3997.

[CIT0006] Li H, Handsaker B, Wysoker A, Fennell T, Ruan J, Homer N, Marth G, Abecasis G, Durbin R 2009 The sequence alignment/map format and SAMtools. Bioinformatics. 25:2078–2079. 19505943 10.1093/bioinformatics/btp352 PMC2723002

[CIT0007] Luo R, Liu B, Xie Y, Li Z, Huang W, Yuan J, et al. 2012 Soapdenovo2: an empirically improved memory-efficient short-read de novo, assembler. Giga Sci. 1:1–6. 10.1186/2047-217X-1-18 PMC3626529 23587118

[CIT0008] Stamatakis A 2014 RAxML version 8: a tool for phylogenetic analysis and post-analysis of large phylogenies. Bioinformatics. 30:1312–1313. 24451623 10.1093/bioinformatics/btu033 PMC3998144

